# Supplementary material for: Preliminary Characterization of Triatomine Bug Blood Meals on the Island of Trinidad Reveals Opportunistic Feeding Behavior on Both Human and Animal Hosts
Source: Trop Med Infect Dis. 2020 Nov 4;5(4):166. doi: 10.3390/tropicalmed5040166 (PMC7709638; doi:10.3390/tropicalmed5040166)
Supplement: Supplementary file 1 [file tropicalmed-05-00166-s001.zip › Hylton et al Supplementary Material.docx]

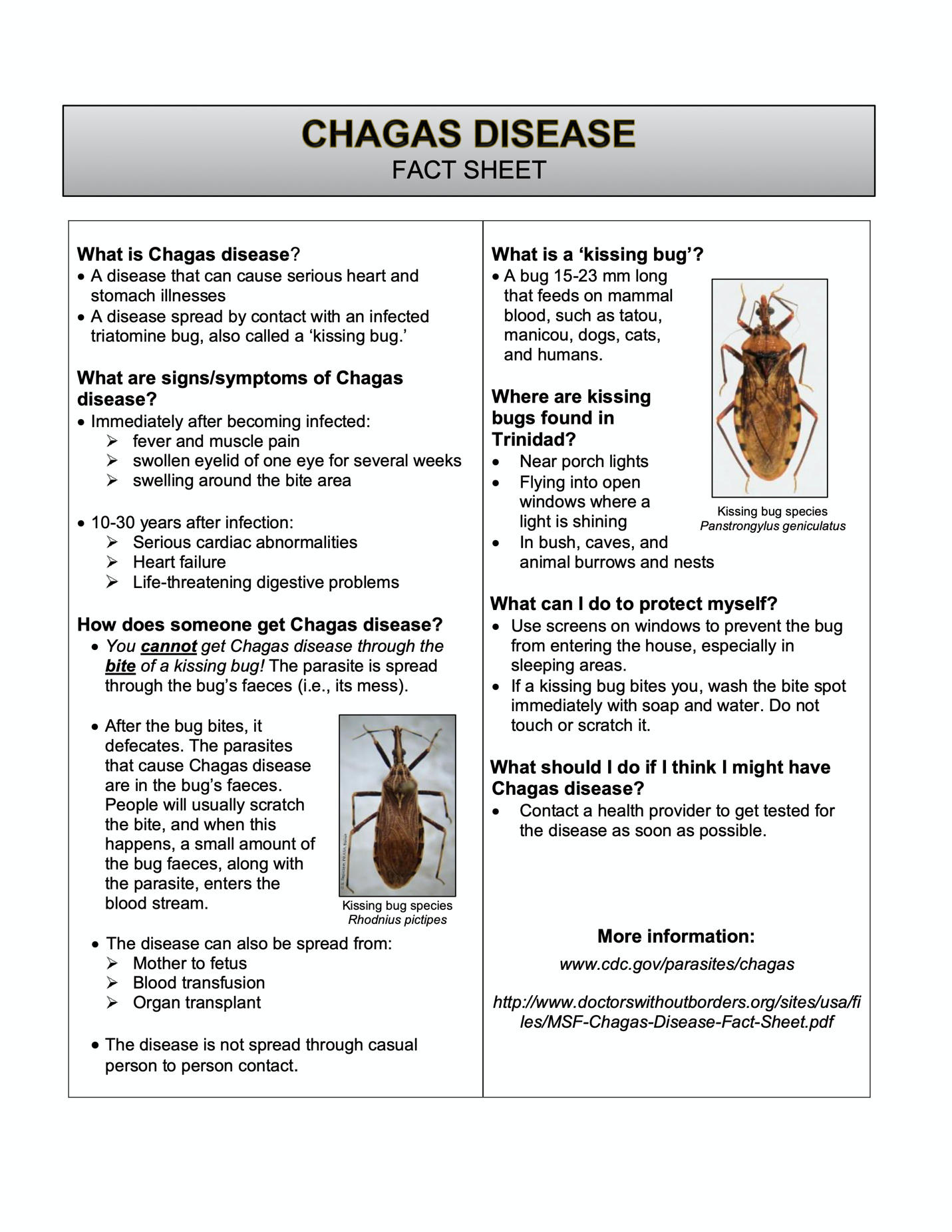
**Figure S1.** Chagas disease information pamphlet given out during this study.

| **Table 1.** Triatomine Bug Sample Site Details. | | | | | |
| --- | --- | --- | --- | --- | --- |
| **Region** | **Village** | **Land use type** | **Sampling spot** | **Collection**  **Method** | **Bugs**  **(y/n)** |
| Northern coast | Blanchieusse | Human use | Home | Community Collection | y |
| Northern range | Champs Fleur | Human use | Trees adjacent to large chicken coop (100+ birds) on a farm with a human residence | Mouse-baited trap | n |
|  | Santa Cruz | Human use | Inside the dryer | Community collection | y |
| Northeast coast | Matura | Forested | Cocorite palms in nature reserve | Mouse-baited trap | n |
|  |  | Mixed | Opossum nest (unsure if occupied) in shrubbery adjacent to a road and a farm | Mouse-baited trap | n |
|  |  | Mixed | Abandoned house in with bat colony in an abandoned cocoa plantation; human residence located 100 ft down the road | Mouse-baited trap | n |
|  |  | Human use | Bat colony in large shed adjacent to human home | Mouse-baited trap | n |
|  |  | Human use | In the kitchen and outside stairs of human home in abandoned cocoa plantation | Community collection | y |
| Central range | Coal Mine | Human use | External home walls | Manual | y |
|  |  | Human use | Artificial light on patio of human home | Mouse-baited trap | y |
|  |  | Human use | Wood pile adjacent to human home | Mouse-baited trap | n |
|  |  | Human use | Tree where chickens roost, 10 ft from human home | Mouse-baited trap | n |
|  |  | Human use | Chicken resting area under human home | Mouse-baited trap | n |
|  |  | Mixed | Deep hole with bats roosting; cocorite trees in forested land adjacent to residential area | Mouse-baited traps | n |
|  | Mount Harris | Human use | Artificial light on ranger station | Mouse-baited trap | y |
|  |  | Human use | Artificial light on external wall of human home | Mouse-baited trap | n |
|  | Plum Road | Human use | Rat nest adjacent to human home (unclear if occupied) | Mouse-baited trap | n |
|  | Tamana Hills | Mixed | Cocorite palms in forested site used for logging | Mouse-baited trap | n |
|  | Tunapuna | Human use | Mount St. Benedict Abbey | Community collection | n |
|  | Valencia | Forested | Cocorite trees | Mouse-baited trap | n |
| Southeast coast | Guayaguayare | Human use | Cocorite palms in plantain farm | Mouse-baited trap | n |

**Publisher’s Note:** MDPI stays neutral with regard to jurisdictional claims in published maps and institutional affiliations.

| 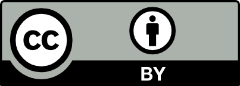 | © 2020 by the authors. Submitted for possible open access publication under the terms and conditions of the Creative Commons Attribution (CC BY) license (http://creativecommons.org/licenses/by/4.0/). |
| --- | --- |
